# Case of Suppuration of the Lungs, with Empyema, Successfully Treated by Paracentesis of the Thorax, and the Internal Use of the Acetate of Lead

**Published:** 1820-02

**Authors:** D. Harke

**Affiliations:** Odessa, in Russia.


FOR THE LONDON MEDICAL AND PHYSICAL JOURNAL.

***Case of Suppuration of the Lungs, with Empyema, successfully treated by Paracentesis of the Thorax, and the internal use of the Acetate of Lead.*** By Dr. D. HARKE, of Odessa, in Russia.

A MAN, forty-five years of age, of a weak constitution, and subject to morbid affections of the chest, became ill, after having been exposed for a considerable time to wet and cold. When I first visited him, he had all the symptoms of advanced phthisis: his pulse was febrile; he had a violent cough; expectoration of matter, of a bad quality; nocturnal sweats; darting pains in the chest; œdema of the feet; anorexia, dyspepsia, &c. For three days past, he had complained of a painful tumor in the left hypochondre, in which an evident fluctuation could be perceived. I had an emolient cataplasm applied to this, and I directed the patient to lie only on the affected side. As all the medicines which had been employed had produced no benefit, and every thing led me to suspect the existence of disorganization of the lungs, and, consequently, to consider the case as a desperate one, I determined to employ the acetate of

---

lead,* and directed half a grain of it to be taken night and morning, with three grains of sugar of milk. A very restricted diet was also prescribed. The patient passed two nights entirely without sleep, with continual cough, copious expectoration, and colliquative sweats. Some days afterwards, I opened the chest, between the sixth and seventh ribs, by an incision with a lancet; and above half a pint of pus, of a yellowish colour, rushed with violence from the thoracic cavity. The wound was lightly covered with a compress, so as to leave a free passage to the pus. The patient was in a state of great depression, and the expectoration was very abundant. He continued to take the acetate of lead. On the ninth day, in the evening, he became very inquiet, complaining of darting pains in the chest; and he expectorated suddenly, after a fit of violent coughing, an enormous quantity of extremely fetid pus. Was this the consequence of the bursting of a vomica? This conjecture appears to be correct, because no more pus was evacuated after this time. The ensuing night was passed in a very disturbed state; the cough was frequent, and the fever violent; but the expectoration was moderated. The use of the acetate of lead was continued. On the tenth day, the patient was more tranquil, the pulse was slower, and the cough more tolerable. On the 15th, he had a little sleep; the fever and the night sweats diminished; a little pus was still passed from the wound; the patient experienced no pain in coughing. About the thirtieth day, the fever disappeared; the cough and the expectoration continued; but the strength of the patient began to increase. The wound, although still open, gave vent to hardly any pus. Besides the acetate of lead, I then ordered him to take half a cupful of a strong decoction of cinchona twice a day. The 37th, the wound was not larger than a pea, and covered with a thin membrane. From this time, the acetate of lead was taken only once a day, and the decoction of cinchona four times. On the 45th day, the wound was cicatrized, and the health of the patient re-established, except that he was yet very weak. I then omitted the use of the acetate of lead, and substituted the jelly of Iceland moss, with sugar and canella, to be given after the cinchona. The use of these remedies was continued for a month, when the patient was perfectly well.*

---

* Dr. Harke had already found this medicine productive of extraordinary benefit in several cases of somewhat analogous diseases of the lungs.—Edit.

† *Russiche Sammlung für Naturwissenschaft und heilkunde.* Herausgegeben von Drs. Crichton, Rehmann, und Burdach, St. Petersburg.

